# *Ex vivo* mapping of enhancer networks that define the transcriptional program driving melanoma metastasis

**DOI:** 10.1002/1878-0261.13485

**Published:** 2023-07-18

**Authors:** Veronica Manicardi, Mila Gugnoni, Elisabetta Sauta, Benedetta Donati, Emanuele Vitale, Federica Torricelli, Gloria Manzotti, Simonetta Piana, Caterina Longo, Francesco Ghini, Alessia Ciarrocchi

**Affiliations:** ^1^ Laboratory of Translational Research Azienda USL‐IRCCS di Reggio Emilia Italy; ^2^ Clinical and Experimental Medicine PhD Program University of Modena and Reggio Emilia Italy; ^3^ Humanitas Clinical and Research Center–IRCCS Milan Italy; ^4^ Pathology Unit Azienda USL‐IRCCS di Reggio Emilia Italy; ^5^ Skin Cancer Unit Azienda USL‐IRCCS di Reggio Emilia Italy

**Keywords:** biological processes, chromatin organization, metastasis, regulatory elements, transcriptional network

## Abstract

Mortality from vmelanoma is associated with metastatic disease, but the mechanisms leading to spreading of the cancer cells remain obscure. Spatial profiling revealed that melanoma is characterized by a high degree of heterogeneity, which is established by the ability of melanoma cells to switch between different phenotypical stages. This plasticity, likely a heritage from embryonic pathways, accounts for a relevant part of the metastatic potential of these lesions, and requires the rapid and efficient reorganization of the transcriptional landscape of melanoma cells. A large part of the non‐coding genome cooperates to control gene expression, specifically through the activity of enhancers (ENHs). In this study, we aimed to identify *ex vivo* the network of active ENHs and to outline their cooperative interactions in supporting transcriptional adaptation during melanoma metastatic progression. We conducted a genome‐wide analysis to map active ENHs distribution in a retrospective cohort of 39 melanoma patients, comparing the profiles obtained in primary (*N* = 19) and metastatic (*N* = 20) melanoma lesions. Unsupervised clustering showed that the profile for acetylated histone H3 at lysine 27 (H3K27ac) efficiently segregates lesions into three different clusters corresponding to progressive stages of the disease. We reconstructed the map of super‐ENHs (SEs) and cooperative ENHs that associate with metastatic progression in melanoma, which showed that cooperation among regulatory elements is a mandatory requirement for transcriptional plasticity. We also showed that these elements carry out specialized and non‐redundant functions, and indicated the existence of a hierarchical organization, with SEs on top as masterminds of the entire transcriptional program and classical ENHs as executors. By providing an innovative vision of how the chromatin landscape of melanoma works during metastatic spreading, our data also point out the need to integrate functional profiling in the analysis of cancer lesions to increase definition and improve interpretation of tumor heterogeneity.

## Graphical abstract

This study aimed to unveil the cooperative network among distal elements, either by local proximity or by long‐range interactions, that sustains the transcriptional plasticity required during progression and metastatic dissemination of melanoma. The findings suggest a hierarchical organization of the regulatory landscape placing super‐enhancers on top as master regulators and classical enhancers as executors of the entire transcriptional program.

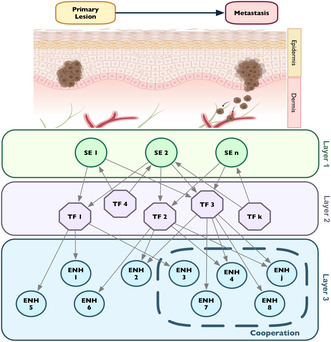

**Abbreviations**
ChIP: chromatin immunoprecipitation
ChIP‐seq: chromatin immunoprecipitation followed by sequencing
DM: distant metastasis
EMT: epithelial‐to‐mesenchymal transition
ENH: enhancer
GO: gene ontology
PT: primary tumor
RE: regulatory element
SE: super‐enhancer
SNP: single nucleotide polymorphism
TF: transcription factor

## 1 Introduction

Cutaneous melanoma is the most aggressive form of skin cancer [1, 2]. Morbidity and mortality are associated with metastatic disease. Despite the large effort developed in melanoma research in the past years, we still do not know the molecular determinants required to eventually establish overt melanoma metastases. A link between development and cancer has been postulated. Factors that are crucial during embryogenesis are often hijacked during cancer progression. This may be particularly relevant in melanoma [3]. During embryogenesis, melanocyte precursors migrate from the neural crest to the epidermis, where they differentiate into mature melanocytes [4]. This plasticity and migratory capacity are retained throughout adult life, since melanocytes have to be constantly replaced, starting from melanoblasts that originate in the hair bulb and migrate to the epidermis to complete their differentiation [5].

Both embryogenesis and cancer progression are established through an elaborate succession of signals that define complex and precise patterns of gene expression. Besides, the precise spatiotemporal expression of a gene needs a continuous and widespread regulatory landscape involving a specific genomic architecture and the hierarchical interaction of multiple interspersed regulatory elements (REs). More than 80% of non‐coding genome is engaged in gene expression regulation and single nucleotide polymorphisms (SNPs) associated with diseases by Genome Wide Association Studies (GWAS) are enriched within non‐coding functional elements. This indicates that sequence alterations in these REs are capable of altering their functionality leading to aberrant cell behavior [6].

A large part of the transcription regulatory function of the non‐coding genome resides within enhancers (ENHs), which are cis‐acting DNA elements working by recruiting transcription factors (TFs) according to highly specific rules [7, 8, 9]. The tissue‐ and time‐specific availability of TFs dictates the way ENHs can be activated. In turn, their activation leads to the correct execution of the genome transcriptional function.

With highly irregular length, ENHs function regardless of their orientation and at varying distances from their target [10].

Their systematic study by genome‐wide chromatin profiling and chromatin interaction mapping revealed that ENHs outnumber genes by at least an order of magnitude, implying not only that multiple ENHs can converge on the regulation of a single gene but also that a single ENH may simultaneously boost transcription of multiple targets to synchronize the transcriptional effects [11]. Indeed, many multimodal cooperative dynamics among these elements have been demonstrated, that mostly are not conserved across different cell types, underlying a stringent functional specificity. The most efficient and characterized form of ENHs cooperation is represented by super‐ENHs (SEs) which are large clusters of closely spaced ENHs, of an average length of 8.7 kbp [12], characterized by massive enrichment of active chromatin markers such as H3K4me1 and H3K27ac and high occupancy of master TFs and RNA polymerase II. Bringing together multiple elements, SEs exponentially increase the expression of target genes bursting transcription efficiency while preventing potential negative perturbations [12, 13, 14]. As such, SEs appear to be of particular importance in highly dynamic processes like embryogenesis and cancer to support the expression of those genes particularly important for the entire process. In cancer, these elements are established during transformation by DNA sequence, structure, or functional alterations, to specifically sustain the expression of driver genes to which cancer cells become addicted and rely on progression [15, 16]. However, the genome is not just a mono‐dimensional entity but occupies a 3D space [17]. Thus, intersperse classical ENHs, even if located at great distance on the linear sequence, can be brought together by chromatin folding organizing long‐range cooperation within SE‐like units that maximize their functional interplay.

Furthermore, ENHs and SEs are highly plastic entities whose existence and genomic distribution change in space and time in a cell‐specific manner [18].

Following the hypothesis that the ability of melanoma cells to grow into overt metastasis relies on the execution of precise patterns of gene expression, in this work we aimed to define the specific network of cooperative ENHs and SEs that sustains these programs. To map these elements, we used deep chromatin profiling starting from human melanoma samples and a combination of bioinformatics and predictive tools to reconstruct the hierarchical organization of the regulatory landscape associated with melanoma metastasis and the network of TFs that foster it.

## 2 Materials and methods

### 2.1 Patients' cohort

A retrospective series of patients‐derived fresh‐frozen 19 primary and 20 metastatic melanoma tissues from the research Biobank of AUSL‐IRCCS Institute were used for the analysis. Samples were collected from July 2014 to March 2019. For three patients both primary and metastatic tissues were available. In all except one primary tumor, prognostic data were available, mainly dimensions, Breslow thickness, Clark level, mitotic activity, and clinical follow‐up (Table S1).

### 2.2 ChIP and ChIP‐sequencing

Tissues were homogenized with Dounce homogenizer in 1% PBS, then crosslinked for 15 min with 1% formaldehyde, lysed, and sonicated for 12 cycles (30″ ON, 30″ OFF) using Bioruptor Pico Sonicator (Diagenode, Liege, Belgium), to obtain 100–200 bp chromatin fragments. Chromatin was precipitated overnight using Dynabeads Protein G magnetic beads (Thermo Fisher Scientific, Waltham, MA, USA) and 3 μg of anti‐H3K27ac (Abcam, Trumpington, UK). One percent of total chromatin was used as input. Samples were quantified at Qubit (Thermo Fisher Scientific) and the quality was evaluated by Bioanalyzer (Agilent, Santa Clara, CA, USA). The NEBNext Ultra II DNA Library Prep Kit for Illumina (New England BioLabs, Ipswich, MA ,USA) was used following manufacturer's instructions, using 5–20 ng ChIP DNA as starting material. Samples were sequenced on Illumina NextSeq500 high‐output cartridge (single‐stranded, read length 75 bp‐1 × 75).

### 2.3 ChIP‐Seq data processing

ChIP‐Seq data processing was performed as previously described [19]. Briefly, sequencing quality was assessed using the fastqc v0.11.8 software (www.bioinformatics.babraham.ac.uk/projects/fastqc/), showing on average a Phred score per base > 30.5 in each sample. Raw sequenced reads were aligned to the Human Reference Genome (GRCh38) using bowtie2 (version 2.3.4.3) [20]. Reads were processed to remove duplicates and unmapped reads. H3K27ac‐enriched regions were identified by comparing the ChIP samples to the input samples using macs2 peak caller (version 2.1.2) [21] with broad peaks settings. Quality analysis using ENCODE ChIP‐seq guidelines [22] was performed on all samples. In particular, depth of sequencing, library complexity, number of peaks, and strand cross‐correlation were evaluated for sample filtering. After quality filtering, 17 primary and 12 metastases were selected for downstream analysis. Significant peaks (*q*‐value < 0.05) were exploited for assessing differential binding by comparing primary versus metastatic samples using diffbind package (version 2.13.1) [23, 24]. The consensus peakset was obtained by selecting only peaks that were detected in at least two samples (minOverlap = 2). Differential bound sites (DBs) were annotated to the corresponding gene by ChIPseeker using the nearest‐neighbor approach. Pathway enrichment analysis on identified genes was performed (Reactome pathways and Gene Ontology—Biological Process were used as reference), considering as a significance threshold a *q*‐value< 0.05 (*P*‐value adjusted by multiple testing, Benjamini–Hochberg correction).

### 2.4 RNA‐sequencing

Patients‐derived fresh‐frozen tissues of the above‐mentioned retrospective cohort were homogenized in TRIzol reagent, and total RNA was extracted following manufacturer's protocol. RNA quality was assessed by Bioanalyzer RNA 6000 pico assay (Agilent). RNA‐seq libraries were obtained starting from 100 ng of total RNA following Illumina Stranded TotalRNA Prep Ligation with Ribo‐zero Plus protocol (Illumina, San Diego, CA, USA). Sequencing was performed using Illumina NextSeq500 high‐output cartridge (double‐stranded, read length 75 bp‐2 × 75 cycles).

### 2.5 RNA‐Seq data processing

Sequencing quality was assessed using the fastqc v0.11.8 software (www.bioinformatics.babraham.ac.uk/projects/fastqc/), showing on average a Phred score per base > 30 in each sample. Paired‐end reads were aligned to the human reference transcriptome (GRCh38, Gencode release 30) using star [25] version 2.7, and gene abundances were estimated with rsem algorithm (v1.3.1). Raw counts were normalized with deseq2 r package [26], then used to assess the expression of SEs‐ and ENHs‐associated genes and to plot the expression level of the predicted TFs in the AUSL‐IRCCS cohort.

### 2.6 Identification of SEs and ENHs

SEs and classical ENHs were called based on their input‐normalized H3K27ac signal intensity in 12.5 kb stitched regions performed by ROSE algorithm [12, 14], using ±3 Kb as promoter region. Genes putative targets of these REs were assigned using the geneMapper function within ROSE method considering all putative targets (closest, overlapping, and proximal). Pathway enrichment analysis on REs‐associated genes was performed (Gene Ontology—Biological Process was used as reference) considering a significance threshold of 0.1 on *P*‐value corrected for multiple testing using Benjamini–Hochberg's method. The network representation of cooperative ENHs target genes enriched in epithelial‐to‐mesenchymal transition and angiogenesis processes was retrieved from string (v11.5) through cytoscape (v3.7.1).

TFClass database was downloaded through rTRM R package and used to classify TFs associated with SEs.

### 2.7 Prediction of SEs and ENHs upstream regulators

The lists of genes associated with the REs were used to predict putative TFs‐regulating melanoma‐associated SEs and ENHs, respectively. The prediction was performed on TRRUST and TRANSFAC&JASPAR databases through enrichr r package [27, 28, 29]. TFs significantly enriched (*q*‐value < 0.1) were further validated by FIMO algorithm [30] to find significant enrichment of their DNA‐binding motifs in SEs and ENHs regions respectively. HOCOMOCO and JASPAR were used as reference motifs databases and a *q*‐value threshold of 0.1 was set to filter the significant motif occurrences. For the ENHs, only genes controlled by at least five ENHs were used for upstream regulators prediction, then the corresponding REs regions were selected and searched for TFs motifs. Bedtools [31] shuffle function was applied on human reference genome (GRCh38/hg38 assembly) to extract random regions with the following setting: ‐noOverlapping, ‐chrom, and ‐excl. The motif search previously described was also performed on these random regions. The statistical significance of TF‐binding sites enrichment was assessed by Chi‐squared test comparing differences in motifs occurrence in REs and background regions. Only TFs with *q*‐value < 0.1 (*P*‐value adjusted by multiple testing, Benjamini–Hochberg correction) and enriched in DM‐associated REs were selected.

### 2.8 Statistical analysis

In each analysis, the *P*‐value was adjusted by the Benjamini–Hochberg method. Comparison between Clusters 1 and 2 for different clinical variables was made by Fisher's exact test (Table 1). Pairwise Wilcoxon test was applied to analyze differences in the expression level of genes under the control of different REs.

**Table 1:** Distribution of PT in Clusters 1 and 2 based on clinical features associated with melanoma aggressiveness.

	Cluster 1 (*N* = 7)	Cluster 2 (*N* = 8)	Total (*N* = 15)	*P*‐value
Vertical growth				
Absent	6 (85.7%)	3 (37.5%)	9 (60.0%)	0.119
Present	1 (14.3%)	5 (62.5%)	6 (40.0%)	
Invasion level				
Clark IV	6 (85.7%)	2 (25.0%)	8 (53.3%)	0.041
Clark V	1 (14.3%)	6 (75.0%)	7 (46.7%)	
Tumor thickness				
Breslow ≤ 4 mm	6 (85.7%)	1 (12.5%)	7 (46.7%)	0.01
Breslow > 4 mm	1 (14.3%)	7 (87.5%)	8 (53.3%)	

### 2.9 Ethics declarations

Each patient who participated in the study provided written informed consent for the biological studies. This study was authorized by the local Ethics Committee (Comitato Etico dell'Area Vasta Emilia Nord; protocol number 2021/0150624 approved on 01/12/2021) and conducted according to Helsinki Declaration.

## 3 Results

### 3.1 Chromatin landscape predicts clinical behavior of melanoma

**Fig. 1:**
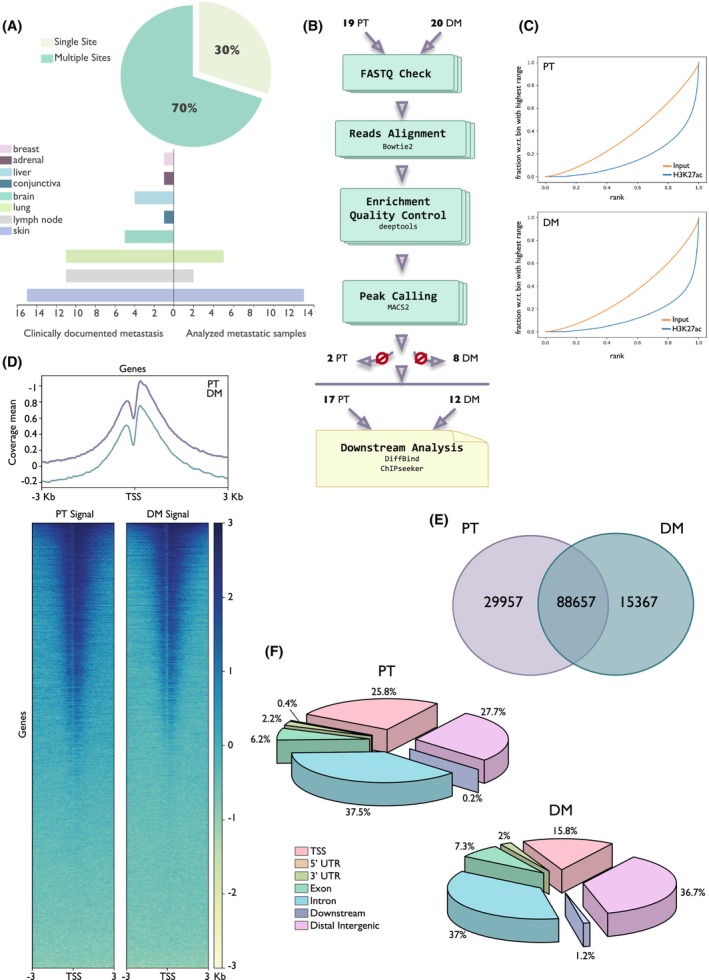
Chromatin landscape in melanoma. (A) Pie chart showing the percentage of distant metastasis (DM) patients with single or multiple metastasis sites (top). Distribution of all clinically documented metastatic sites for DM patients included in the study (bottom left) and distribution of the 20 patients' derived distant metastasis samples that were profiled (bottom right). (B) Flow chart of ChIP‐seq data collection and analysis. Number of starting, dropped, and selected patients are specified. (C) Fingerprint plot of H3K27ac ChIP‐seq libraries and the corresponding input in a representative primary tumor (PT, top) and distant metastasis (DM, bottom) sample. (D) Distribution of H3K27ac ChIP‐seq signal at TSS (±3 kb) in representative primary tumor (PT, lilac, left) and distant metastasis (DM, cadet blue, right) sample. Each profile was normalized on its input. (E) Venn diagram showing the overlap between the H3K27ac‐binding sites of the consensus peakset in primary tumors (PT) and distant metastasis (DM) samples. (F) Genomic distribution of H3K27ac peaks differentially associated with primary tumors (PT) versus distant metastasis (DM) samples. Pie chart shows the percentage of peaks falling within transcription start site of genes (TSS), gene exons (Exon), gene introns (Intron), regions within 300 bp from gene end (downstream), 5′ UTR and 3′ UTR of genes, and intergenic regions (distal intergenic).

To map REs active in metastatic melanoma, we performed chromatin immunoprecipitation (ChIP)‐sequencing analysis for H3K27ac in 39 melanoma patient‐derived tissue samples collected in our Institution. Of these, 19 were primary melanoma (PT) and 20 were melanoma metastasis (DM) from different sites, in particular skin, lung, and lymph nodes (Fig. 1A, Table S1). Through fingerprint plot, we confirmed the deviation of each ChIP curve from its input (Fig. 1C). Also, H3K27ac signal distribution around TSS [−3 kb; +3] of genes was evaluated (Fig. 1D). After quality controls, 17 PTs and 12 DMs were considered adequate for further analysis (Fig. 1B–D, Table S2). The consensus peakset was obtained by selecting only peaks that were consistently detected in at least two samples, resulting in a total of 133 981 peaks identified. Of these, 29 957 peaks were exclusive of PTs and 15 367 were exclusive of DMs (Fig. 1E). Figure 1F shows the genomic distribution of consensus H3K27ac peaks in both sets of samples. Peaks were enriched on promoters (25.8% for PTs and 15.8% for DMs) and distal intergenic elements (27.7% in PTs and 36.7% in DMs). In both groups, 37% of H3K27ac peaks fell within intronic regions, whereas around 7% was enriched in gene exons. In DMs, peaks annotated at distal intergenic regions increased, whereas a slight loss in peaks associated with TSS regions was observed compared to PT samples. To evaluate the variation in the binding pattern between PTs and DMs, a differential binding analysis was performed. We took advantage of DiffBind R package to outline differences in H3K27ac‐binding events between the two groups of samples. The differential analysis was performed by applying DESeq2 method and considering a *q*‐value threshold of 0.1 to identify differentially H3K27ac‐enriched sites. These regions underwent a peak‐to‐target assignment to associate them with the closest gene, thus predicting the putative target gene of each H3K27ac peak. We identified 1051 genes under the regulation of these differential elements, and GO enrichment analysis showed that these genes are implicated in cancer progression‐related processes like regulation of cell motility, cell–cell or cell–matrix interaction, and cell structure (Fig. S1A).

**Fig. 2:**
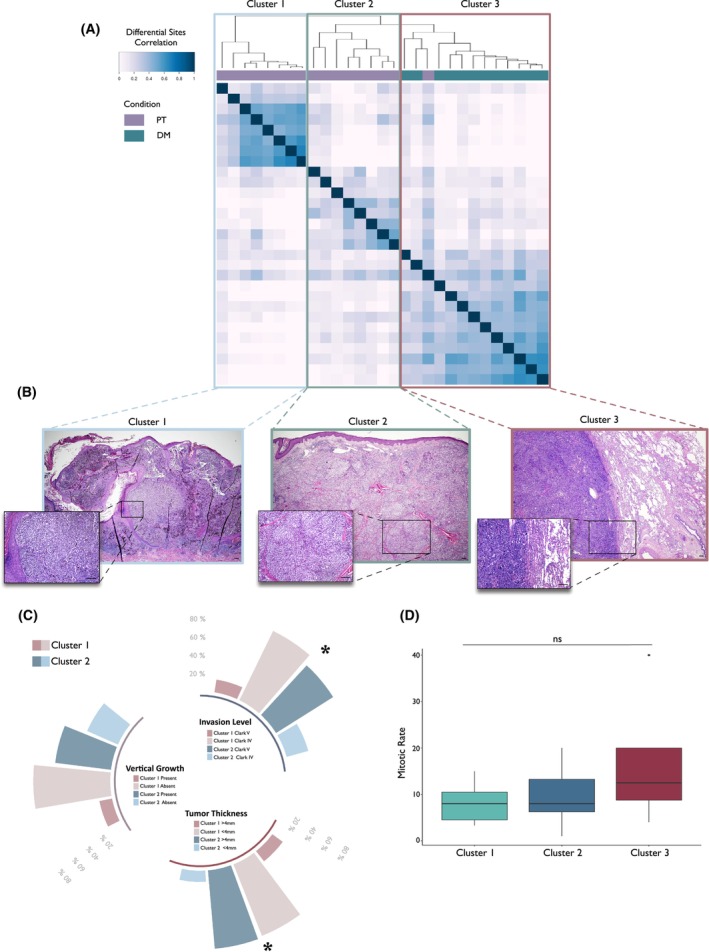
Chromatin status predicts clinical behavior of melanoma. (A) Unsupervised clustering analysis of primary tumor (PT) and distant metastasis (DM) samples based on the H3K27Ac profiles. Clusters are highlighted with rectangles: light blue = PT cluster 1, olive = PT cluster 2, dark red = DM, cluster 3. (B) Hematoxylin and eosin (H&E) staining of representative samples from PT c1, PT c2, and DM. Scale bar 200 μm. (C) Histogram of primary tumor clinical features associated with melanoma aggressiveness. Patients' distribution is shown as a percentage of the total of the corresponding cluster. Invasion level = Clark level and tumor thickness = Breslow index. *Fisher's exact test *P* < 0.05. (D) Boxplots showing the distribution of the mitotic rate of the lesions in each identified cluster. The boxes represent the interquartile range (IQR) with whiskers extending to minimum and maximum data points within 1.5 times the IQR. Median values are shown as lines inside the boxes. Outliers are shown as individual data points beyond the whiskers. ns = Fisher's exact test *P* > 0.05.

Unsupervised clustering analysis based on H3K27ac profiles segregated samples into three different groups. PT samples were distributed into two clusters (Clusters 1 and 2), while all DM samples except one were comprised in Cluster 3 (Fig. 2A). Next, we integrated ChIP‐seq profiling with patient clinical data to define the identified clusters. Noticeably, within the PT samples, clustering distribution correlated with tri‐dimensional growth and invasiveness of the lesions (Fig. 2B). In particular, Cluster 1 was enriched in lesions with a lower Breslow index (< 4 mm) and inferior Clark. Also, a non‐significant association between Cluster 2 and vertical growth was observed (Fig. 2C, Table 1). As shown in Fig. 2D, there was a trend in the increase in mitotic rate from Cluster 1 to Cluster 3, further supporting the idea that Cluster 1 and Cluster 2 PTs have different aggressiveness potential and behavior. For the following analyses, we focused on PTs in Cluster 2 (PTs c2) and DMs since PTs c2 showed a higher aggressive potential.

Together these data indicate that the landscape of the transcriptionally active REs across the genome is associated with different clinical behaviors of melanoma and predicts aggressiveness.

### 3.2 Metastasis‐associated SEs govern a central core of melanoma essential TFs

**Fig. 3:**
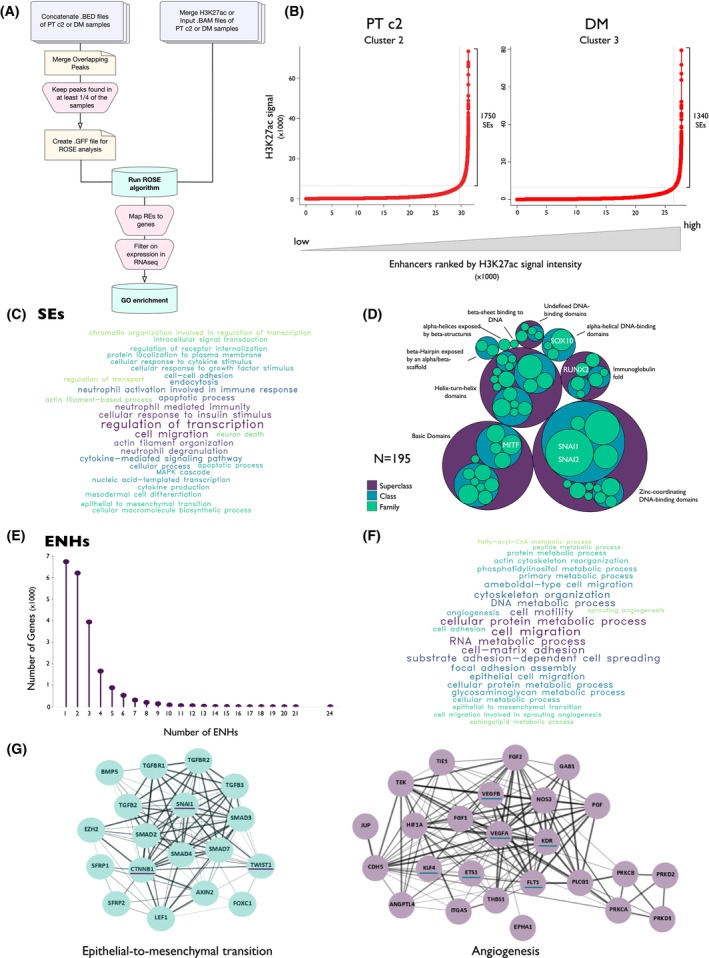
SEs and cooperative ENHs exist in a hierarchical interplay to regulate pro‐metastatic gene programs in DM. (A) Schematic representation of data preparation and ROSE analysis. (B) Super‐enhancers (SEs) and enhancers (ENHs) ranking on input‐normalized H3K27ac signal intensity in stitched regions performed by ROSE on primary tumors from Cluster 2 (PT c2) and distant metastases (DM). (C, F) GO enrichment analysis of SEs (C) and ENHs (F) predicted targets. Size of words is proportional to the −log10(*q*‐value). Pathways shown were filtered for *q*‐value < 0.1. (D) Classification of SEs‐associated transcription factors (TFs) based on their DNA‐binding domains. TFs were hierarchically grouped into family (green), class (cyan), and super‐class (purple). Names of super‐classes are shown on plot. Genes described in the text are reported in white within their family. (E) Lollipop plot of the number of ENHs associated with the number of their predicted target genes. (G) STRING protein–protein interaction prediction of ENHs target genes enriched in epithelial‐to‐mesenchymal transition and angiogenesis pathways. Genes cited in the text are underscored.

To map the cooperative ENHs network associated with metastatic behavior in melanoma, we used the ROSE [12, 14] algorithm that based on the H3K27ac enrichment level discriminates SEs from classical ENHs (Fig. 3A).

A total of 1750 SEs and 29 654 classical ENHs were predicted for PTs c2 (Fig. 3B). In which, 614 of 1750 (35%) SEs were specific to PT c2 samples and were associated with 1014 genes, all of which were expressed at mRNA level based on RNAseq data. As for PT‐associated classical ENHs, 7925 of 29 654 (26.7%) were specific for this group and 5612 genes were identified as putative targets of these ENHs and their expression was confirmed by RNA‐seq. Genes controlled by PT c2‐associated SEs were enriched in processes like cytoskeleton organization, stress fiber assembly, and endothelial cell migration (Fig. S1B). On the other hand, ENHs‐associated gene signature in PT c2 group was mainly involved in neuron development, Wnt signaling pathway, and protein phosphorylation and modification processes (Fig. S1C). A similar analysis was performed on elements that were unique to DM samples (Fig. S1D,E).

Contrary to chromatin profiling, transcriptional profile of the same patients' cohort (AUSL‐IRCCS) and the TCGA Skin Cutaneous Melanoma (TCGA‐SKCM) project was not able to clearly and completely segregate PT c2 and DM samples, even if considering only SEs and ENHs target genes (Fig. S2).

Therefore, to have a comprehensive overview of the processes associated with metastatic development in melanoma, we focused on the overall chromatin landscape of DMs. Considering all active elements detected in these samples, 1340 SEs and 26 469 classical ENHs were predicted by ROSE algorithm (Fig. 3B). Among these elements, 210 (16%) SEs and 4154 (15.7%) ENHs were specific to DM group.

A total of 3418 genes were predicted as potential targets of SEs. Ranking them on the number of associated SEs, we observed that 88% of these genes were uniquely associated with a single element. 71.2% (*N* = 2433) of the SEs predicted target genes were confirmed by RNA‐seq to be expressed at the mRNA level in our samples. GO analysis showed that these genes were enriched in several biological pathways, among which five major functional categories could be identified (Fig. 3C, Table S3): transcriptional regulation, immune response/immunity, cell movement, cytoskeleton organization, and cell–cell interaction. Noticeably, 195 TFs were identified among the SEs predicted target genes (Fig. 3D), including SOX10 and MITF, master drivers of the melanocytic lineage, and well‐established transcriptional dependency of melanoma [32, 33] (Table S4). We also noticed that transcriptional regulators of EMT including SNAI1, SNAI2, and RUNX2 were present in this list, supporting the hypothesis that the phenotypical plasticity gained through this transdifferentiation process concurs with melanoma aggressiveness.

### 3.3 Cooperative ENHs concur with the regulation of executive pro‐metastatic gene programs

Considering DM samples, 20 898 genes were predicted to be associated with the 26 469 classical ENHs identified. We ranked these genes for the number of classical ENHs that converged on their regulation (Fig. 3E) and we observed that 67.7% (*N* = 14 155) of them were associated with more than one ENH, and 11.2% of genes (*n* = 2340) were predicted to be associated with five or more ENHs. We hypothesized that the convergence of multiple regulatory elements on the same gene could underline functional cooperation among distal ENHs for their regulation. Thus, we focused on the genes associated with five or more elements and found that 1750 of 2340 (74.8%) genes predicted to be controlled by cooperative ENHs were confirmed to be expressed in our samples. Interestingly, GO enrichment analysis highlighted that these genes were enriched in cell adhesion and movement, epithelial‐to‐mesenchymal transition, metabolic pathways, and angiogenesis, all crucial processes in the metastatic progression of cancer (Fig. 3F, Table S5). Among the predicted targets of the cooperative ENHs network associated with DMs, we found some of the principal mediators of TGFβ and WNT signaling, major triggers of EMT, including the TFs CTNNB1, TWIST1, and SNAI1 [34, 35] (Fig. 3G). This is particularly relevant since heteromorphic shape and structure as well as the ability to switch between an epithelial and a mesenchymal‐like state re part of the dynamic plasticity that has been discovered as distinctive trait of aggressive melanoma [36, 37]. In addition, VEGFA and B, their receptors KDR and FLT1, and downstream TFs, including KLF4 and ETS1 [38], were found in this analysis as targets of cooperative ENHs, in line with the pivotal role of angiogenesis in promoting cancer metastatic spreading [39].

Taken together, this evidence indicates that SEs and classical ENHs concur with the execution of the metastatic program by governing two distinct layers of the hierarchical network.

ENHs cooperation ensures a higher transcriptional performance during the metastasization process. SEs have been described as elements that boost transcription of pivotal cancer genes of several orders of magnitude as compared to regular ENHs.

To evaluate the impact of SEs on gene expression program of DM samples, we assessed whether the expression of the predicted SEs proximity genes was significantly different from the one of genes associated with ENHs. We separated REs into three main groups: classical ENHs and cooperative ENHs and SEs. We extracted the list of genes predicted under the regulation of each group, then filtered genes on RNA‐seq data to remove those that were not expressed in AUSL‐IRCCS cohort. Taking into account the average expression of each gene among DM samples, we evaluated the expression level of the gene signature associated with each group of REs. Significant differences in expression levels were observed for genes under the control of different elements (Fig. S3A). In particular, SEs‐associated genes were significantly more expressed than genes governed by classical ENHs. In addition, predicted targets of cooperative ENHs were on average more expressed than those under classical ENHs control but less expressed than SEs‐associated genes. These data strengthen the idea that chromatin landscape displays a hierarchical organization during melanoma progression and further highlights how distal or proximal cooperation among REs is required to finely tune gene expression during complex biological processes.

### 3.4 A core of TFs controls the transcriptional program associated with metastasis in melanoma

Aberrant gene expression in cancer is governed by oncogenic TFs, whose binding to DNA drives activation of regulatory regions and orchestrates transcription in space and time. Thus, to functionally interpret the transcriptional landscape that supports DM, we intended to define the core of TFs governing its activation.

**Fig. 4:**
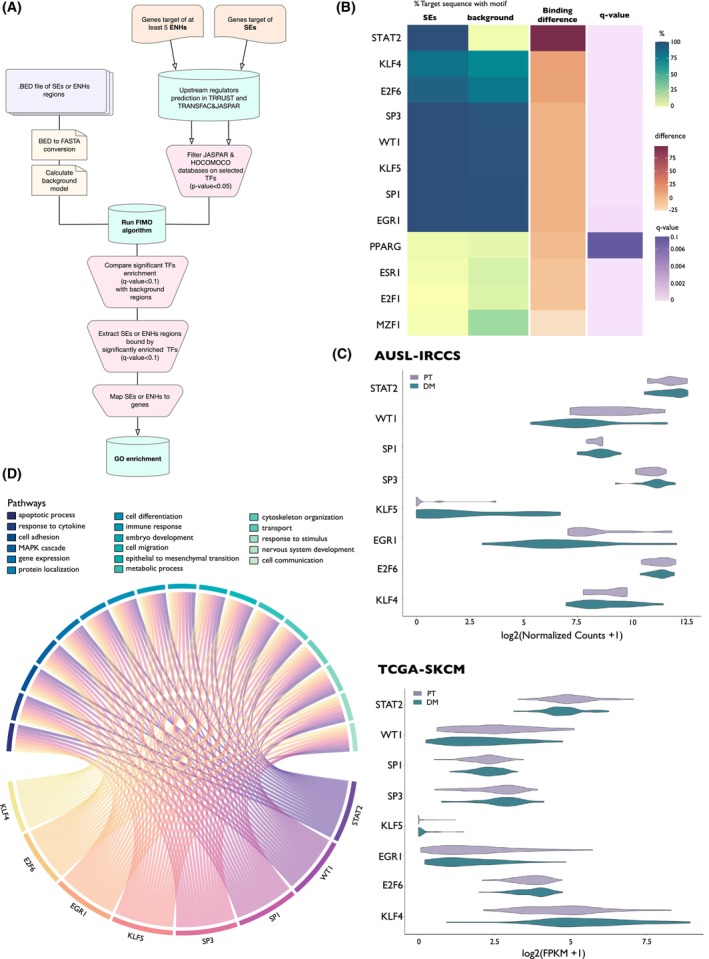
A core of TFs drives SE‐associated transcriptional program. (A) Schematic representation of the motif search in super‐enhancers (SEs) and enhancers (ENHs) regions. (B) Motif enrichment analysis in SEs regions in distant metastasis (DM) samples. Heatmap represents the percentage of regions predicted to be bound by each transcription factor (TF) (SEs and background), difference in binding percentage between analyzed regions (binding difference), and significance of comparison (*q*‐value). (C) Violin plot of the expression of the enriched TFs in the AUSL‐IRCCS patients' cohort (top) and the TCGA‐SKCM dataset (bottom). (D) Chord diagram showing pathway GO macro‐categories in which the genes controlled by SEs‐binding TFs are enriched in DMs. Width of TFs nodes is proportional to the number of DM‐associated SEs controlled by each TF.

To this aim, a series of sequential prediction approaches were combined (Fig. 4A). First, we used two separate databases (TRRUST and TRANSFAC&JASPAR) to predict potential upstream transcriptional regulators in parallel on the list of target genes associated with either SEs or more than five ENHs (cooperative ENHs). Only significantly enriched TFs were selected (*P*‐value < 0.05). Next, starting from H3K27ac ChIP‐seq profiles of our samples, we used FIMO [30] to search for their binding motifs and validate their potential interaction with the identified SEs and ENHs, respectively. For both Res, the significant TFs (*q*‐value < 0.1) were ranked on difference of binding between REs and background regions. *Q*‐value < 0.1 and binding difference > 0 were considered to select TFs significantly enriched in REs as compared to background (Table S6). Noticeably, STAT2, WT1, SP1, SP3, KLF5, EGR1, E2F6, and KLF4 scored as top TFs in SEs regulation (Fig. 4B), while only EGR1 motifs were significantly enriched in ENHs regions (Fig. S3B, Table S7). Strikingly, STAT2 was greatly enriched within SEs and it showed the highest difference in binding when comparing its occurrence in SEs and background regions. This is in line with the fact that immunity and immune response regulation scored as top biological pathways under SEs regulation in our analysis. Intriguingly, STAT2 was also the most expressed TF both in our sample set and in the TCGA‐SKCM dataset (Fig. 4C). No differences in the expression of all these factors was observed between PT and DM, suggesting that variation in the accessibility of the regulatory regions accounts for the activation of metastatic pathways. Chord plot in Fig. 4D shows the influence of each TF on the macro‐categories of the biological processes controlled by SEs.

## 4 Discussion

In spite of being one of the most investigated and profiled type of tumor, many aspects of melanoma biology remain elusive, among which are drivers and mechanisms supporting the transition toward a metastatic phenotype. Many factors concur with this lack. First, the fact that melanoma progression does not follow a linear evolution in which the ability to metastasize is acquired over time as the result of accumulating genetic alterations [3]. Recent single‐cell and spatial profiling studies revealed that melanoma cells exist in multiple phenotypical states and that can switch among these very dynamically [37, 40, 41, 42, 43]. This plasticity likely accounts for a large part of melanoma aggressive behavior but still does not explain how and why some lesions get to play stronger than others [44]. Laying behind this plasticity there is a prompt and efficient reorganization of the gene expression program supporting the adaptation of the cellular phenotype during transitions. To get insights into the chromatin dynamics that govern such processes here, we employed a functional approach to profile, *ex vivo*, changes in the transcriptional status of the REs during melanoma metastatic spreading. We showed that the genome‐wide distribution of the active chromatin marker H3K27ac predicts clinical behavior of the lesions, clustering the melanoma biopsies in three unsupervised groups corresponding to progressive stages of the disease. These results even if limited by the modest number of analyzed samples represent a significant advantage in the understanding of the molecular basis of this disease.

First, to the best of our knowledge, this is one of the first studies that profile the chromatin landscape of melanoma starting from *ex vivo* samples.

Second, the functional approach that we employed allowed us to overcome the limitation of a purely descriptive transcriptomic profile producing a more faithful representation of the molecular dynamics supporting progression that recapitulate the actual clinical condition.

The systematic mapping of ENHs by genome‐wide chromatin profiling across cell types revealed that these elements outnumber genes of at least an order of magnitude [6, 45], underlining the existence of a complex and multimodal cooperative interaction that dictate the execution of precise transcriptional programs [46]. SEs represent the most evident example of such cooperation [8]. Being organized as linear stretches of single ENHs close within a defined genomic region, SEs are organized as modular elements, in which each regulatory unit interacts with each other to maximize the transcriptional effect limiting signal fluctuation [12, 13, 14]. SEs are not invariant elements but their existence is highly cell context dependent. It is for this reason that SEs in cancer are deputed to the transcriptional control of functional essential genes to which cancer cells are addicted. Thus, reconstructing the network of SEs that governs cancer progression is emerging as a potent tool to identify new functional dependencies that represent new vulnerabilities of these lesions.

Our analysis, which is one of the first mapping SEs organization in melanoma patient samples showed that in the context of metastatic melanoma, this infrastructure serves to support three major biological aspects. Of particular interest is the fact that transcription regulation was the top‐scoring category in this analysis. A total of 195 TFs were predicted to be under the regulation of these elements including major, already established transcriptional dependency of melanoma (SOX10 and MITF) [47, 48, 49] or factors like SNAI1 and SNAI2 that drive the transition toward the mesenchymal phenotype. Noticeably, the mesenchymal‐like state has emerged as one of the major transcriptional conditions in which melanoma cells exist *in vivo*, and it has been linked to increased migratory potential and resistance to therapies.

By using a two steps prediction approach, we predicted TFs with a potential function of SEs upstream regulators, ranking them based on binding difference. With great interest, we found STAT2 as top scoring in this analysis, followed by major oncogenic TFs including KLF4 and E2F6.

Primary function of STAT2 is to regulate immune activation in response to IFN and other pro‐inflammatory cytokines [50, 51]. Indeed, among the genes predicted to be under the SEs' control many were involved in regulation of immune response and IFN signaling. This is not surprising, since the interplay between melanoma cells and the immune microenvironment at the metastatic site seems to be of crucial relevance in the metastatic behavior of these lesions [52]. Indeed, according to the parallel evolution model, melanoma cells depart early from the primary lesion and disseminate to distant sites where they persist quiescently until they acquire the ability to form overt metastasis. Systemic immune modulatory signals seem to be crucial in defining the timing of this event since it often happens once the primary lesion has been surgically removed. Acting downstream to these signals and being positioned basically on each of the identified SEs, STAT2 (and likely other members of this family) candidates to be a master regulator of the metastatic switch in melanoma. STAT2 protein levels have been shown to be higher in melanoma than in normal skin and to correlate with increased melanoma cell proliferation [53]. In line with this evidence, STAT2 and its companion IRF9 were found to mediate resistance to BRAFi [54] and to be associated with amoeboid phenotype in melanoma [55].

We are aware that this analysis is exploratory and limited by the lack of functional data and information on chromatin topological organization in these samples. However, our analysis showed that convergence of different elements on the same genes is a major feature of melanoma chromatin landscape with 38% (*N* = 7940) of the classical ENHs predicted genes being associated with more than two elements. Exacerbating this cooperation, we focused on genes that were predicted to be under the regulation of five or more ENHs. Surprisingly, GO analysis revealed that these genes belonged primarily to EMT and angiogenesis regulation. These biological properties, central in the factual execution of the metastatic spreading, do not overlap with the ones that emerged under the regulation of SEs, thus indicating a functional specialized and non‐redundant function of these diverse elements in melanoma.

## 5 Conclusions

Taken together, our data provide an innovative vision of how the chromatin landscape of melanoma works during metastatic spreading. The model emerging from our data seems to indicate a hierarchical organization of the transcriptional landscape of melanoma with SEs on top of this organization as masterminds of the entire transcriptional program and classical ENHs as executors.

Also, our data further underline the evidence that cooperation among single REs is a mandatory requirement of transcriptional plasticity, being necessary to tune the adaptation of cancer cell phenotype in response to the many stimuli during progression. Finally, our data point to the need of integrating a functional point of view in profiling cancer heterogeneity to increase definition and improve interpretation.

## Conflict of interest

The authors declare no conflict of interest.

## Author contributions

VM, ES, and FT contributed to data analysis. AC and MG contributed to the conceptualization, design, and supervision of the project. SP and CL provided melanoma samples and clinical data. BD, EV, GM, and FG contributed to the experiments. All authors have read and agreed to the published version of the manuscript.

## Supporting information


**Fig. S1.** GO enrichment analysis of multiple lists of genes.
**Fig. S2.** Relationship between samples based on gene expression.
**Fig. S3.** Expression levels of distal elements' targets and motif enrichment on cooperative enhancers.
**Table S1.** Descriptive statistics of primary melanoma patients of AUSL‐IRCCS cohort.
**Table S2.** List of ChIP‐seq samples.
**Table S3.** List of Gene Ontology—Biological Process enriched for SEs‐associated genes.
**Table S4.** Classification of the 195 transcription factors target of SEs on TFClass database.
**Table S5.** List of Gene Ontology—Biological Process enriched for ENHs‐associated genes.
**Table S6.** Motif search of predicted TFs‐binding motifs within DM‐associated SEs.
**Table S7.** Motif search of predicted TFs‐binding motifs within DM‐associated cooperative ENHs.

## Data Availability

ChIP‐seq and RNA‐seq data generated and analyzed during the current study are available in the ArrayExpress repository with the following accession code: E‐MTAB‐12244 for ChIP‐seq dataset (https://www.ebi.ac.uk/arrayexpress/experiments/E‐MTAB‐12244) and E‐MTAB‐12649 for RNA‐seq dataset (https://www.ebi.ac.uk/arrayexpress/experiments/E‐MTAB‐12649).
