# Supplementary material for: Ex vivo mapping of enhancer networks that define the transcriptional program driving melanoma metastasis
Source: Mol Oncol. 2023 Jul 18;17(12):2728–42. doi: 10.1002/1878-0261.13485 (PMC10701766; doi:10.1002/1878-0261.13485)
Supplement: Supplementary file 1 — Fig. S1. GO enrichment analysis of multiple lists of genes. Fig. S2. Relationship between samples based on gene expression. Fig. S3. Expression levels of distal elements' targets and motif enrichment on cooperative enhancers. Table S1. Descriptive statistics of primary melanoma patients of AUSL‐IRCCS cohort. Table S2. List of ChIP‐seq samples. Table S3. List of Gene Ontology—Biological Process enriched for SEs‐associated genes. Table S4. Classification of the 195 transcription factors target of SEs on TFClass database. Table S5. List of Gene Ontology—Biological Process enriched for ENHs‐associated genes. Table S6. Motif search of predicted TFs‐binding motifs within DM‐associated SEs. Table S7. Motif search of predicted TFs‐binding motifs within DM‐associated cooperative ENHs. [file MOL2-17-2728-s001.pdf]

FIGURE S1

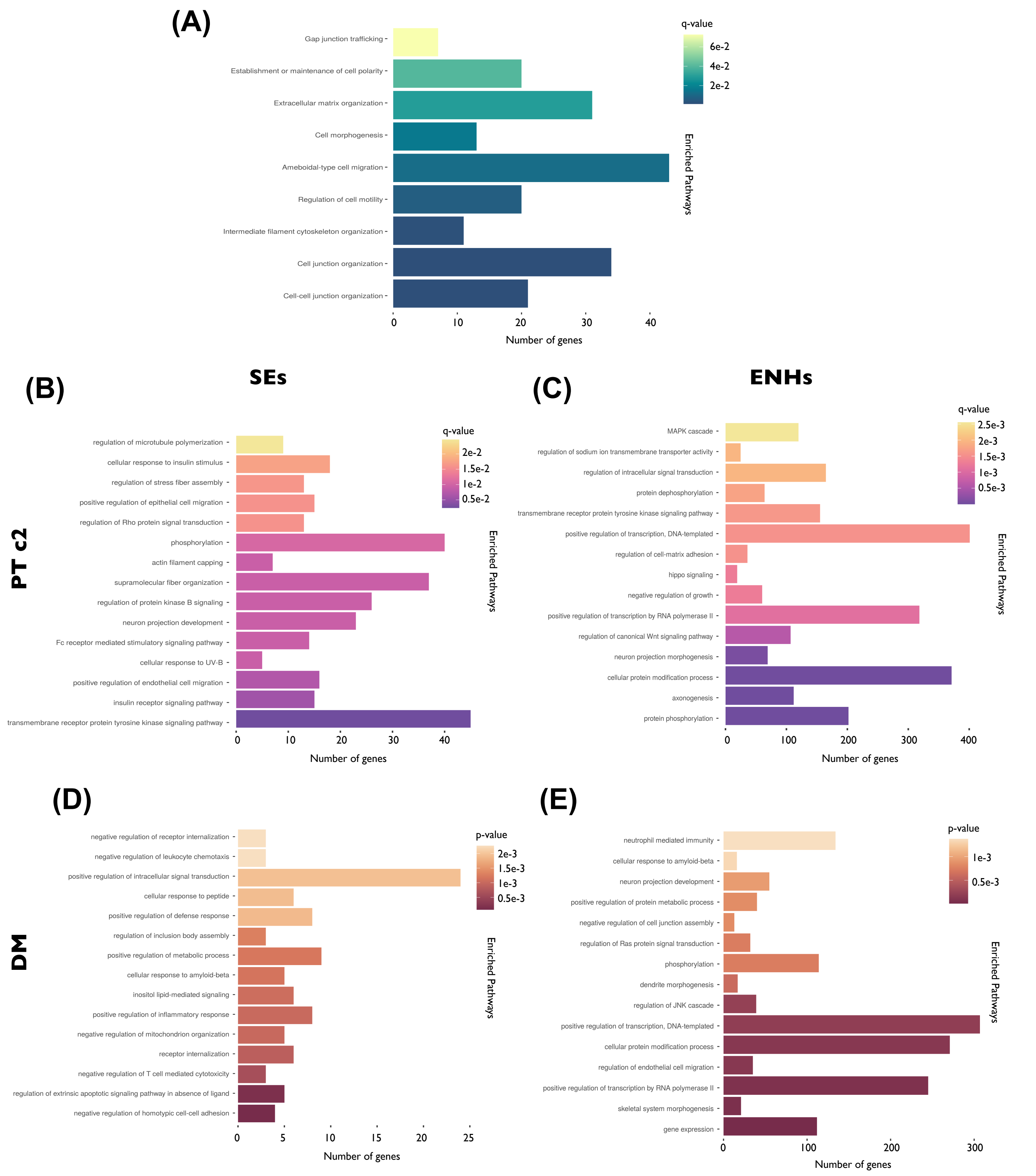

Figure S1 *GO enrichment analysis of multiple list of genes* (A) GO enrichment of genes predicted as targets of H3K27ac differentially active regions. Bar dimension is proportional to the number of genes enriched in the pathway, bar color is based on pathway significance level (q-value). (B-C) GO enrichment of genes controlled by primary tumor cluster 2 (PT c2)-specific SEs (B) or ENHs (C). Bar dimension is proportional to the number of genes enriched in the pathway, bar color is based on pathway significance level (q-value). (D-E) GO enrichment of genes controlled by distant metastasis (DM)-specific SEs (B) or ENHs (C). Bar dimension is proportional to the number of genes enriched in each pathway, bar color is based on pathway significance level (p-value or q-value).

FIGURE S2

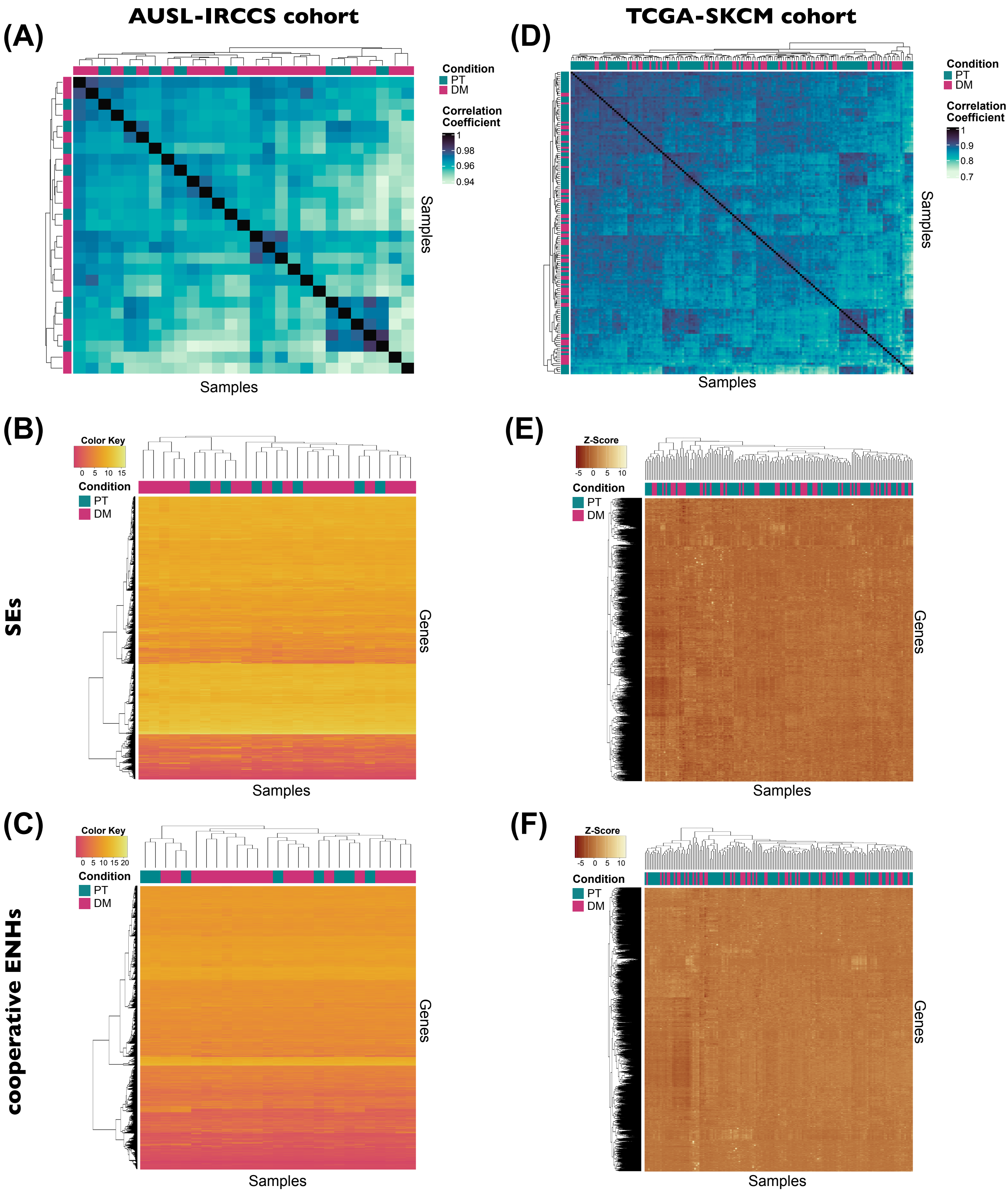

Figure S2 *Relationship between samples based on gene expression* (A) Spearman correlation of AUSL-IRCCS samples on whole expression matrix. Regularized logarithm transformation was applied on the raw expression matrix as suggested by DESeq2 manual for data visualization. (B-C) Heatmap of genes associated with distant metastases' SEs (B) or cooperative ENHs (C) in AUSL-IRCCS cohort. Yellow=high expression, Red=low expression. Expression data were row scaled and centered for graphical purposes. (D) Spearman correlation of TCGA-SCKM samples on whole expression matrix. TCGA-SKCM expression data were downloaded through TCGABiolinks as FPKM, then transformed to log2(FPKM+1) for data visualization. (E-F) Heatmap of genes associated with distant metastases' SEs (E) or cooperative ENHs (F) in TCGA-SKCM cohort. Yellow=high expression, Red=low expression. PT = Primary Tumor, DM=distant metastasis.

FIGURE S3

(A)

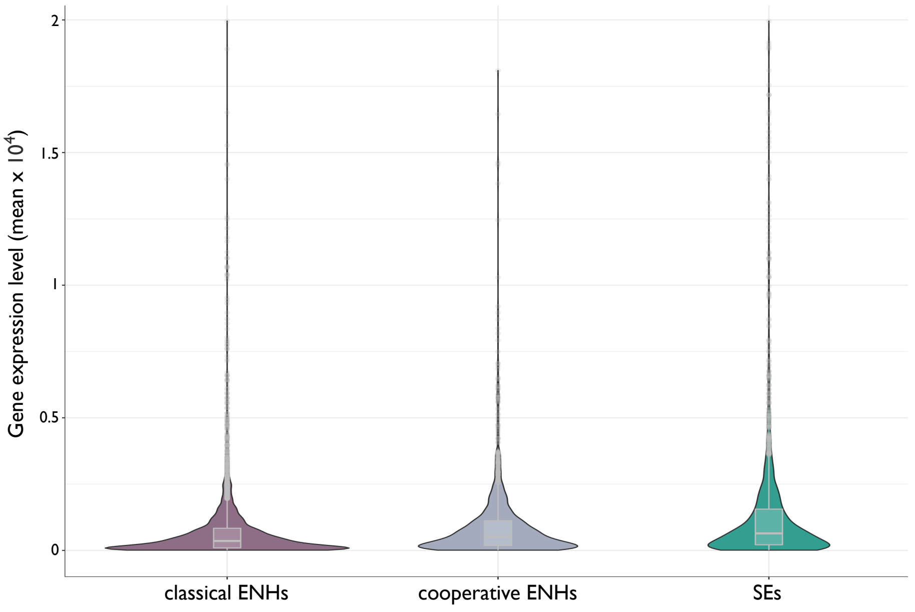

	classical ENHs	cooperative ENHs
**cooperative ENHs**	<2e-16	-
**SEs**	<2e-16	9.4e-08

(B)

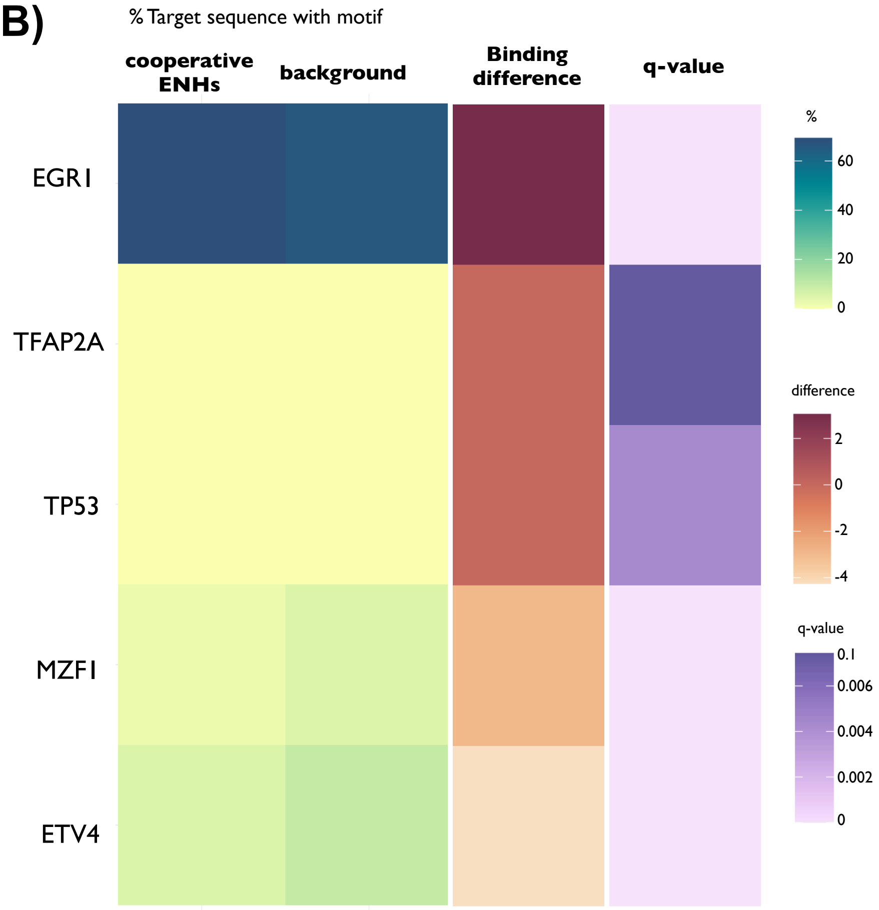

Figure S3 *Expression level of distal elements' targets and motif enrichment on cooperative enhancers* (A) Distribution of averaged expression level of genes associated with classical ENHs, cooperative ENHs and SEs in distant metastasis samples. Y-axis was cut to 20000 for graphical purposes. Results of the pairwise Wilcoxon test applied on each comparison are shown in the table on the right. (B) Motif enrichment analysis in cooperative ENHs regions in distant metastasis samples. Heatmap represents the percentage of regions predicted to be bound by each transcription factor (TF) (cooperative ENHs and background), difference in binding percentage between analyzed regions (binding difference) and significance of comparison (q-value).

Table S1 Descriptive Statistics of primary melanoma patients of AUSL-IRCCS cohort

Clinical Characteristics	Patients (n = 19), N (%) or Value
**Sex**	
F	5 (26.3%)
M	14 (73.7%)
**Age** (years) (mean±SD)	68.8±15.1
**Size** (mm) (mean±SD)	29.4±13.3
**BRAF**	
wild type	6 (31.6%)
BRAFV600E	5 (26.3%)
missing	8 (42.1%)
**Mitotic Activity** (index) (mean±SD)	10.5±9.2
**Clark level**	
IV	10 (52.6%)
V	8 (42.1%)
missing	1 (5.3%)
**Breslow index**	
< 2 mm	2 (10.5%)
> 2 mm, <=4	6 (31.6%)
> 4 mm	10 (52.6%)
missing	1 (5.3%)
**Site of lesion**	
abdomen	1 (5.3%)
back	6 (31.6%)
face	2 (10.5%)
flank	1 (5.3%)
forearm	1 (5.3%)
hand	1 (5.3%)
leg	1 (5.3%)
nail	1 (5.3%)
scalp	3 (15.8%)
thigh	1 (5.3%)
missing	1 (5.3%)

Table S2 List of ChIP-seq samples

SampleName	H3K27ac Reads	INPUT Reads	Number of Significant Peaks
BTC02	45022168	24161183	72561
BTC09	42210492	68329878	48030
BTC106	40064441	62102131	21967
BTC13	37911760	37912109	138921
BTC143	44971175	43319270	3362
BTC15	8328662	10571960	5
BTC19	38513391	16970708	0
BTC20	6526503	5008209	10349
BTC24	97960765	44526127	31352
BTC26	31159683	48918550	95394
BTC37	32757906	42186715	43528
BTC45	24059747	41456606	65755
BTC55	36952204	38015140	34802
BTC57	47720665	47800661	156099
BTC59	31472226	39706071	86452
BTC88	41373378	37400786	36045
BTC92	39955103	43770260	17633
BTC28	36146598	34975088	23806
BTC85	40837435	37740596	82813
MET08	40492327	52640415	9665
MET101	40810134	24366609	0
MET103	50133420	25999219	0
MET105	57021833	28572654	0
MET114	32739997	62927535	97592
MET124	54909265	30164295	54891
MET162	48081177	39547660	0
MET29	48367348	44322306	44008
MET34	34567691	47083740	73
MET52	33608199	44518499	42453
MET68	56429447	42383169	44209
MET73	45920507	44034791	56604
MET79	35843139	38409465	18624
MET84	38746377	38720651	0
MET95	38732634	41362496	90201
MET102	37363429	40066785	0
MET110	50379728	48154930	1
MET113	36741429	51295739	27157
MET121	39065447	45600786	110022
MET129	41554188	52981198	106572

Samples in red were excluded from the analysis because of low quality.

Table S3 List of Gene Ontology - Biological Process enriched for SEs-associated genes.

Pathway	Gene Ratio	Number of genes	p-value	q-pvalue
regulation of transcription	0.18	174	1.09E-08	5.37E-05
cell migration	0.21	85	3.83E-07	4.72E-04
cellular response to insulin stimulus	0.27	35	3.04E-06	2.50E-03
neutrophil mediated immunity	0.19	93	6.31E-06	4.44E-03
actin filament organization	0.25	38	9.70E-06	5.84E-03
neutrophil degranulation	0.19	91	1.07E-05	5.84E-03
apoptotic process	0.22	51	1.52E-05	6.82E-03
neutrophil activation involved in immune response	0.19	91	1.49E-05	6.82E-03
cytokine-mediated signaling pathway	0.18	111	1.75E-05	7.18E-03
endocytosis	0.33	20	2.02E-05	7.66E-03
cellular process	0.18	100	7.14E-05	2.51E-02
cell-cell adhesion	0.64	7	8.25E-05	2.71E-02
MAPK cascade	0.27	25	1.06E-04	3.12E-02
nucleic acid-templated transcription	0.18	84	1.14E-04	3.12E-02
cellular response to growth factor stimulus	0.23	36	1.32E-04	3.43E-02
cellular response to cytokine stimulus	0.18	86	1.58E-04	3.58E-02
cytokine production	0.19	64	1.54E-04	3.58E-02
protein localization to plasma membrane	0.28	22	1.60E-04	3.58E-02
mesodermal cell differentiation	0.67	6	1.95E-04	3.60E-02
apoptotic process	0.17	123	1.97E-04	3.60E-02
regulation of receptor internalization	0.58	7	1.77E-04	3.60E-02
epithelial to mesenchymal transition	0.33	14	2.85E-04	5.02E-02
cellular macromolecule biosynthetic process	0.17	94	3.16E-04	5.03E-02
intracellular signal transduction	0.18	78	3.09E-04	5.03E-02
neuron death	0.32	15	3.00E-04	5.03E-02
actin filament-based process	0.27	20	3.29E-04	5.07E-02
chromatin organization involved in regulation of transcription	0.54	7	3.44E-04	5.13E-02
regulation of transport	0.25	23	4.41E-04	6.04E-02

Table S4 Classification of the 195 transcription factors target of SEs on TFclass database.

Gene	Family name	Class name	Superclass name
AHR	PAS domain factors	Basic helix-loop-helix factors (bHLH)	Basic domains
ALX1	Paired-related HD factors	Homeo domain factors	Helix-turn-helix domains
ARID1A	ARID-related factors	ARID domain factors	Helix-turn-helix domains
ARID1B	ARID-related factors	ARID domain factors	Helix-turn-helix domains
ARID5B	ARID-related factors	ARID domain factors	Helix-turn-helix domains
ARNT2	PAS domain factors	Basic helix-loop-helix factors (bHLH)	Basic domains
ATF4	ATF-4-related factors	Basic leucine zipper factors (bZIP)	Basic domains
ATOH8	Tal-related factors	Basic helix-loop-helix factors (bHLH)	Basic domains
BACH1	Jun-related factors	Basic leucine zipper factors (bZIP)	Basic domains
BCL3	Ankyrin domain-only factors	Rel homology region (RHR) factors	Immunoglobulin fold
BCL6	More than 3 adjacent zinc finger factors	C2H2 zinc finger factors	Zinc-coordinating DNA-binding domains
BHLHE40	Hairy-related factors	Basic helix-loop-helix factors (bHLH)	Basic domains
BHLHE41	Hairy-related factors	Basic helix-loop-helix factors (bHLH)	Basic domains
BNC2	Factors with multiple dispersed zinc fingers	C2H2 zinc finger factors	Zinc-coordinating DNA-binding domains
CEBPB	C/EBP-related	Basic leucine zipper factors (bZIP)	Basic domains
CEBPD	C/EBP-related	Basic leucine zipper factors (bZIP)	Basic domains
CIC	SOX-related factors	High-mobility group (HMG) domain factors	Other all-alpha-helical DNA-binding domains
CREB3L2	CREB-related factors	Basic leucine zipper factors (bZIP)	Basic domains
CSRNP1	CSRNP factors	AXUD/CSRNP domain factors	Yet undefined DNA-binding domains
DDIT3	C/EBP-related	Basic leucine zipper factors (bZIP)	Basic domains
DLX3	NK-related factors	Homeo domain factors	Helix-turn-helix domains
DNMT3A	GATA-type zinc fingers	Other C4 zinc finger-type factors	Zinc-coordinating DNA-binding domains
E2F4	E2F-related factors	Fork head / winged helix factors	Helix-turn-helix domains
E2F7	E2F-related factors	Fork head / winged helix factors	Helix-turn-helix domains
EGR3	Three-zinc finger Krüppel-related factors	C2H2 zinc finger factors	Zinc-coordinating DNA-binding domains
ELF1	Ets-related factors	Tryptophan cluster factors	Helix-turn-helix domains
EPAS1	PAS domain factors	Basic helix-loop-helix factors (bHLH)	Basic domains
ERF	Ets-related factors	Tryptophan cluster factors	Helix-turn-helix domains
ETS1	Ets-related factors	Tryptophan cluster factors	Helix-turn-helix domains
ETV1	Ets-related factors	Tryptophan cluster factors	Helix-turn-helix domains

ETV3	Ets-related factors	Tryptophan cluster factors	Helix-turn-helix domains
ETV4	Ets-related factors	Tryptophan cluster factors	Helix-turn-helix domains
ETV5	Ets-related factors	Tryptophan cluster factors	Helix-turn-helix domains
ETV6	Ets-related factors	Tryptophan cluster factors	Helix-turn-helix domains
ETV7	Ets-related factors	Tryptophan cluster factors	Helix-turn-helix domains
FOS	Fos-related factors	Basic leucine zipper factors (bZIP)	Basic domains
FOSB	Fos-related factors	Basic leucine zipper factors (bZIP)	Basic domains
FOSL2	Fos-related factors	Basic leucine zipper factors (bZIP)	Basic domains
FOXO3	Forkhead box (FOX) factors	Fork head / winged helix factors	Helix-turn-helix domains
GATAD2A	GATA-type zinc fingers	Other C4 zinc finger-type factors	Zinc-coordinating DNA-binding domains
GLI3	More than 3 adjacent zinc finger factors	C2H2 zinc finger factors	Zinc-coordinating DNA-binding domains
HBP1	SOX-related factors	High-mobility group (HMG) domain factors	Other all-alpha-helical DNA-binding domains
HES1	Hairy-related factors	Basic helix-loop-helix factors (bHLH)	Basic domains
HEY1	Hairy-related factors	Basic helix-loop-helix factors (bHLH)	Basic domains
HEY2	Hairy-related factors	Basic helix-loop-helix factors (bHLH)	Basic domains
HIVEP1	Factors with multiple dispersed zinc fingers	C2H2 zinc finger factors	Zinc-coordinating DNA-binding domains
HIVEP2	Factors with multiple dispersed zinc fingers	C2H2 zinc finger factors	Zinc-coordinating DNA-binding domains
HMG20A	TOX-related factors	High-mobility group (HMG) domain factors	Other all-alpha-helical DNA-binding domains
HMG20B	TOX-related factors	High-mobility group (HMG) domain factors	Other all-alpha-helical DNA-binding domains
HMGA2	HMGA factors	A.T hook factors	beta-Sheet binding to DNA
HOXB2	HOX-related factors	Homeo domain factors	Helix-turn-helix domains
HOXB3	HOX-related factors	Homeo domain factors	Helix-turn-helix domains
HOXB4	HOX-related factors	Homeo domain factors	Helix-turn-helix domains
HOXB5	HOX-related factors	Homeo domain factors	Helix-turn-helix domains
HOXB6	HOX-related factors	Homeo domain factors	Helix-turn-helix domains
HSF4	HSF factors	Heat shock factors	Helix-turn-helix domains
IKZF4	Factors with multiple dispersed zinc fingers	C2H2 zinc finger factors	Zinc-coordinating DNA-binding domains
IRF1	Interferon-regulatory factors	Tryptophan cluster factors	Helix-turn-helix domains
IRF2	Interferon-regulatory factors	Tryptophan cluster factors	Helix-turn-helix domains
IRF4	Interferon-regulatory factors	Tryptophan cluster factors	Helix-turn-helix domains
IRF6	Interferon-regulatory factors	Tryptophan cluster factors	Helix-turn-helix domains
JARID2	ARID-related factors	ARID domain factors	Helix-turn-helix domains
JUNB	Jun-related factors	Basic leucine zipper factors (bZIP)	Basic domains
JUND	Jun-related factors	Basic leucine zipper factors (bZIP)	Basic domains

KDM2A	CpG-binding proteins	CXXC zinc finger factors	Zinc-coordinating DNA-binding domains
KLF10	Three-zinc finger Krüppel-related factors	C2H2 zinc finger factors	Zinc-coordinating DNA-binding domains
KLF13	Three-zinc finger Krüppel-related factors	C2H2 zinc finger factors	Zinc-coordinating DNA-binding domains
KLF3	Three-zinc finger Krüppel-related factors	C2H2 zinc finger factors	Zinc-coordinating DNA-binding domains
KLF6	Three-zinc finger Krüppel-related factors	C2H2 zinc finger factors	Zinc-coordinating DNA-binding domains
KLF7	Three-zinc finger Krüppel-related factors	C2H2 zinc finger factors	Zinc-coordinating DNA-binding domains
KLF9	Three-zinc finger Krüppel-related factors	C2H2 zinc finger factors	Zinc-coordinating DNA-binding domains
LEF1	TCF-7-related factors	High-mobility group (HMG) domain factors	Other all-alpha-helical DNA-binding domains
LRRFIP1	LRRFIP factors	Leucine-rich repeat flightless-interacting proteins	Yet undefined DNA-binding domains
LRRFIP2	LRRFIP factors	Leucine-rich repeat flightless-interacting proteins	Yet undefined DNA-binding domains
LYAR	LYAR-related proteins	C2HC zinc finger factors	Zinc-coordinating DNA-binding domains
LYL1	Tal-related factors	Basic helix-loop-helix factors (bHLH)	Basic domains
MAF	Maf-related factors	Basic leucine zipper factors (bZIP)	Basic domains
MAFF	Maf-related factors	Basic leucine zipper factors (bZIP)	Basic domains
MAFG	Maf-related factors	Basic leucine zipper factors (bZIP)	Basic domains
MAFK	Maf-related factors	Basic leucine zipper factors (bZIP)	Basic domains
MAX	bHLH-ZIP factors	Basic helix-loop-helix factors (bHLH)	Basic domains
MAZ	Factors with multiple dispersed zinc fingers	C2H2 zinc finger factors	Zinc-coordinating DNA-binding domains
MEF2A	Regulators of differentiation	MADS box factors	alpha-Helices exposed by beta-structures
MEF2D	Regulators of differentiation	MADS box factors	alpha-Helices exposed by beta-structures
MITF	bHLH-ZIP factors	Basic helix-loop-helix factors (bHLH)	Basic domains
MLXIP	bHLH-ZIP factors	Basic helix-loop-helix factors (bHLH)	Basic domains
MNT	bHLH-ZIP factors	Basic helix-loop-helix factors (bHLH)	Basic domains
MTA2	GATA-type zinc fingers	Other C4 zinc finger-type factors	Zinc-coordinating DNA-binding domains
MYBL1	Myb/SANT domain factors	Tryptophan cluster factors	Helix-turn-helix domains
MYC	bHLH-ZIP factors	Basic helix-loop-helix factors (bHLH)	Basic domains
NCOR2	Myb/SANT domain factors	Tryptophan cluster factors	Helix-turn-helix domains
NFATC2	NFAT-related factors	Rel homology region (RHR) factors	Immunoglobulin fold
NFE2L1	Jun-related factors	Basic leucine zipper factors (bZIP)	Basic domains
NFE2L2	Jun-related factors	Basic leucine zipper factors (bZIP)	Basic domains
NFE2L3	Jun-related factors	Basic leucine zipper factors (bZIP)	Basic domains
NFIC	Nuclear factor 1	SMAD/NF-1 DNA-binding domain factors	beta-Hairpin exposed by an alpha/beta-scaffold
NFIX	Nuclear factor 1	SMAD/NF-1 DNA-binding domain factors	beta-Hairpin exposed by an alpha/beta-scaffold
NFKBIA	Ankyrin domain-only factors	Rel homology region (RHR) factors	Immunoglobulin fold

NPAS3	PAS domain factors	Basic helix-loop-helix factors (bHLH)	Basic domains
NR1D1	Thyroid hormone receptor-related factors (NR1)	Nuclear receptors with C4 zinc fingers	Zinc-coordinating DNA-binding domains
NR2F2	RXR-related receptors (NR2)	Nuclear receptors with C4 zinc fingers	Zinc-coordinating DNA-binding domains
NR3C1	Steroid hormone receptors (NR3)	Nuclear receptors with C4 zinc fingers	Zinc-coordinating DNA-binding domains
NR4A1	NGFI-B-related receptors (NR4)	Nuclear receptors with C4 zinc fingers	Zinc-coordinating DNA-binding domains
NR4A2	NGFI-B-related receptors (NR4)	Nuclear receptors with C4 zinc fingers	Zinc-coordinating DNA-binding domains
PAX3	Paired plus homeo domain	Paired box factors	Helix-turn-helix domains
PBX2	TALE-type homeo domain factors	Homeo domain factors	Helix-turn-helix domains
PHF5A	PHF5	Uncharacterized	Yet undefined DNA-binding domains
PKNOX2	TALE-type homeo domain factors	Homeo domain factors	Helix-turn-helix domains
PPP1R13L	Ankyrin domain-only factors	Rel homology region (RHR) factors	Immunoglobulin fold
PRDM1	More than 3 adjacent zinc finger factors	C2H2 zinc finger factors	Zinc-coordinating DNA-binding domains
RARA	Thyroid hormone receptor-related factors (NR1)	Nuclear receptors with C4 zinc fingers	Zinc-coordinating DNA-binding domains
RARB	Thyroid hormone receptor-related factors (NR1)	Nuclear receptors with C4 zinc fingers	Zinc-coordinating DNA-binding domains
RARG	Thyroid hormone receptor-related factors (NR1)	Nuclear receptors with C4 zinc fingers	Zinc-coordinating DNA-binding domains
RERE	GATA-type zinc fingers	Other C4 zinc finger-type factors	Zinc-coordinating DNA-binding domains
RFX5	RFX-related factors	Fork head / winged helix factors	Helix-turn-helix domains
RREB1	Factors with multiple dispersed zinc fingers	C2H2 zinc finger factors	Zinc-coordinating DNA-binding domains
RUNX1	Runt-related factors	Runt domain factors	Immunoglobulin fold
RUNX2	Runt-related factors	Runt domain factors	Immunoglobulin fold
RUNX3	Runt-related factors	Runt domain factors	Immunoglobulin fold
RXRG	RXR-related receptors (NR2)	Nuclear receptors with C4 zinc fingers	Zinc-coordinating DNA-binding domains
SMAD3	SMAD factors	SMAD/NF-1 DNA-binding domain factors	beta-Hairpin exposed by an alpha/beta-scaffold
SMAD7	SMAD factors	SMAD/NF-1 DNA-binding domain factors	beta-Hairpin exposed by an alpha/beta-scaffold
SNAI1	More than 3 adjacent zinc finger factors	C2H2 zinc finger factors	Zinc-coordinating DNA-binding domains
SNAI2	More than 3 adjacent zinc finger factors	C2H2 zinc finger factors	Zinc-coordinating DNA-binding domains
SOX10	SOX-related factors	High-mobility group (HMG) domain factors	Other all-alpha-helical DNA-binding domains
SOX13	SOX-related factors	High-mobility group (HMG) domain factors	Other all-alpha-helical DNA-binding domains
SOX15	SOX-related factors	High-mobility group (HMG) domain factors	Other all-alpha-helical DNA-binding domains
SOX4	SOX-related factors	High-mobility group (HMG) domain factors	Other all-alpha-helical DNA-binding domains
SOX5	SOX-related factors	High-mobility group (HMG) domain factors	Other all-alpha-helical DNA-binding domains
SP1	Three-zinc finger Krüppel-related factors	C2H2 zinc finger factors	Zinc-coordinating DNA-binding domains
SP3	Three-zinc finger Krüppel-related factors	C2H2 zinc finger factors	Zinc-coordinating DNA-binding domains
SREBF1	bHLH-ZIP factors	Basic helix-loop-helix factors (bHLH)	Basic domains

STAT5A	STAT factors	STAT domain factors	Immunoglobulin fold
STAT5B	STAT factors	STAT domain factors	Immunoglobulin fold
STAT6	STAT factors	STAT domain factors	Immunoglobulin fold
TADA2B	Myb/SANT domain factors	Tryptophan cluster factors	Helix-turn-helix domains
TBX2	TBX2-related factors	T-Box factors	Immunoglobulin fold
TBX3	TBX2-related factors	T-Box factors	Immunoglobulin fold
TBX4	TBX2-related factors	T-Box factors	Immunoglobulin fold
TBX6	TBX6-related factors	T-Box factors	Immunoglobulin fold
TCF25	Nuclear localized protein 1	Uncharacterized	Yet undefined DNA-binding domains
TEAD1	TEF-1-related factors	TEA domain factors	Helix-turn-helix domains
TFAP2A	AP-2	Basic helix-span-helix factors (bHSH)	Basic domains
TGIF1	TALE-type homeo domain factors	Homeo domain factors	Helix-turn-helix domains
THAP3	THAP-related factors	C2CH THAP-type zinc finger factors	Zinc-coordinating DNA-binding domains
THRA	Thyroid hormone receptor-related factors (NR1)	Nuclear receptors with C4 zinc fingers	Zinc-coordinating DNA-binding domains
TSC22D1	ZIP only	Basic leucine zipper factors (bZIP)	Basic domains
TSC22D3	ZIP only	Basic leucine zipper factors (bZIP)	Basic domains
TSHZ1	HD-ZF factors	Homeo domain factors	Helix-turn-helix domains
VEZF1	Factors with multiple dispersed zinc fingers	C2H2 zinc finger factors	Zinc-coordinating DNA-binding domains
ZBTB2	Factors with multiple dispersed zinc fingers	C2H2 zinc finger factors	Zinc-coordinating DNA-binding domains
ZBTB20	More than 3 adjacent zinc finger factors	C2H2 zinc finger factors	Zinc-coordinating DNA-binding domains
ZBTB38	Factors with multiple dispersed zinc fingers	C2H2 zinc finger factors	Zinc-coordinating DNA-binding domains
ZBTB46	Other factors with up to three adjacent zinc fingers	C2H2 zinc finger factors	Zinc-coordinating DNA-binding domains
ZBTB47	More than 3 adjacent zinc finger factors	C2H2 zinc finger factors	Zinc-coordinating DNA-binding domains
ZBTB48	More than 3 adjacent zinc finger factors	C2H2 zinc finger factors	Zinc-coordinating DNA-binding domains
ZBTB49	More than 3 adjacent zinc finger factors	C2H2 zinc finger factors	Zinc-coordinating DNA-binding domains
ZBTB7A	More than 3 adjacent zinc finger factors	C2H2 zinc finger factors	Zinc-coordinating DNA-binding domains
ZBTB7B	More than 3 adjacent zinc finger factors	C2H2 zinc finger factors	Zinc-coordinating DNA-binding domains
ZEB2	HD-ZF factors	Homeo domain factors	Helix-turn-helix domains
ZFAT	Factors with multiple dispersed zinc fingers	C2H2 zinc finger factors	Zinc-coordinating DNA-binding domains
ZFHX3	HD-ZF factors	Homeo domain factors	Helix-turn-helix domains
ZFP62	More than 3 adjacent zinc finger factors	C2H2 zinc finger factors	Zinc-coordinating DNA-binding domains
ZFP91	More than 3 adjacent zinc finger factors	C2H2 zinc finger factors	Zinc-coordinating DNA-binding domains
ZKSCAN1	More than 3 adjacent zinc finger factors	C2H2 zinc finger factors	Zinc-coordinating DNA-binding domains
ZKSCAN5	Factors with multiple dispersed zinc fingers	C2H2 zinc finger factors	Zinc-coordinating DNA-binding domains

ZNF114	More than 3 adjacent zinc finger factors	C2H2 zinc finger factors	Zinc-coordinating DNA-binding domains
ZNF142	Factors with multiple dispersed zinc fingers	C2H2 zinc finger factors	Zinc-coordinating DNA-binding domains
ZNF217	Factors with multiple dispersed zinc fingers	C2H2 zinc finger factors	Zinc-coordinating DNA-binding domains
ZNF251	Factors with multiple dispersed zinc fingers	C2H2 zinc finger factors	Zinc-coordinating DNA-binding domains
ZNF280A	More than 3 adjacent zinc finger factors	C2H2 zinc finger factors	Zinc-coordinating DNA-binding domains
ZNF280B	More than 3 adjacent zinc finger factors	C2H2 zinc finger factors	Zinc-coordinating DNA-binding domains
ZNF283	More than 3 adjacent zinc finger factors	C2H2 zinc finger factors	Zinc-coordinating DNA-binding domains
ZNF296	Factors with multiple dispersed zinc fingers	C2H2 zinc finger factors	Zinc-coordinating DNA-binding domains
ZNF34	More than 3 adjacent zinc finger factors	C2H2 zinc finger factors	Zinc-coordinating DNA-binding domains
ZNF362	More than 3 adjacent zinc finger factors	C2H2 zinc finger factors	Zinc-coordinating DNA-binding domains
ZNF384	More than 3 adjacent zinc finger factors	C2H2 zinc finger factors	Zinc-coordinating DNA-binding domains
ZNF394	More than 3 adjacent zinc finger factors	C2H2 zinc finger factors	Zinc-coordinating DNA-binding domains
ZNF487	Other factors with up to three adjacent zinc fingers	C2H2 zinc finger factors	Zinc-coordinating DNA-binding domains
ZNF517	More than 3 adjacent zinc finger factors	C2H2 zinc finger factors	Zinc-coordinating DNA-binding domains
ZNF518B	Factors with multiple dispersed zinc fingers	C2H2 zinc finger factors	Zinc-coordinating DNA-binding domains
ZNF652	More than 3 adjacent zinc finger factors	C2H2 zinc finger factors	Zinc-coordinating DNA-binding domains
ZNF687	Factors with multiple dispersed zinc fingers	C2H2 zinc finger factors	Zinc-coordinating DNA-binding domains
ZNF689	More than 3 adjacent zinc finger factors	C2H2 zinc finger factors	Zinc-coordinating DNA-binding domains
ZNF697	Factors with multiple dispersed zinc fingers	C2H2 zinc finger factors	Zinc-coordinating DNA-binding domains
ZNF7	More than 3 adjacent zinc finger factors	C2H2 zinc finger factors	Zinc-coordinating DNA-binding domains
ZNF710	More than 3 adjacent zinc finger factors	C2H2 zinc finger factors	Zinc-coordinating DNA-binding domains
ZNF740	Other factors with up to three adjacent zinc fingers	C2H2 zinc finger factors	Zinc-coordinating DNA-binding domains
ZNF764	More than 3 adjacent zinc finger factors	C2H2 zinc finger factors	Zinc-coordinating DNA-binding domains
ZNF768	More than 3 adjacent zinc finger factors	C2H2 zinc finger factors	Zinc-coordinating DNA-binding domains
ZNF774	More than 3 adjacent zinc finger factors	C2H2 zinc finger factors	Zinc-coordinating DNA-binding domains
ZNF785	More than 3 adjacent zinc finger factors	C2H2 zinc finger factors	Zinc-coordinating DNA-binding domains
ZNF789	More than 3 adjacent zinc finger factors	C2H2 zinc finger factors	Zinc-coordinating DNA-binding domains
ZNF860	More than 3 adjacent zinc finger factors	C2H2 zinc finger factors	Zinc-coordinating DNA-binding domains
ZSCAN21	More than 3 adjacent zinc finger factors	C2H2 zinc finger factors	Zinc-coordinating DNA-binding domains

Table S5 List of Gene Ontology - Biological Process enriched for ENHs-associated genes.

Pathway	Gene ratio	Number of genes	p-value	q-value
cell migration	0.79	324	3.42E-08	5.56E-06
cellular protein metabolic process	0.89	91	2.39E-07	2.50E-05
RNA metabolic process	0.86	115	3.47E-07	3.27E-05
cell-matrix adhesion	0.89	89	4.23E-07	3.86E-05
cell motility	0.87	99	1.46E-06	9.92E-05
substrate adhesion-dependent cell spreading	0.96	46	1.64E-06	1.08E-04
DNA metabolic process	0.80	221	2.66E-06	1.59E-04
cytoskeleton organization	0.86	103	3.04E-06	1.74E-04
focal adhesion assembly	0.93	50	1.09E-05	5.01E-04
epithelial cell migration	0.95	39	1.93E-05	7.92E-04
ameboidal-type cell migration	0.92	48	2.08E-05	8.39E-04
cellular protein metabolic process	0.76	319	2.28E-05	8.91E-04
glycosaminoglycan metabolic process	0.91	49	5.57E-05	1.94E-03
primary metabolic process	0.82	106	2.17E-04	5.35E-03
angiogenesis	0.82	95	3.35E-04	7.60E-03
phosphatidylinositol metabolic process	0.82	92	3.37E-04	7.60E-03
cellular metabolic process	0.89	42	4.49E-04	9.62E-03
actin cytoskeleton reorganization	0.92	34	4.69E-04	9.83E-03
cell adhesion	0.80	107	5.48E-04	1.09E-02
protein metabolic process	0.88	42	1.25E-03	2.00E-02
epithelial to mesenchymal transition	0.82	62	4.25E-03	4.78E-02
cell migration involved in sprouting angiogenesis	0.88	28	8.59E-03	7.90E-02
peptide metabolic process	0.80	66	1.01E-02	8.84E-02
sphingolipid metabolic process	0.78	90	1.03E-02	8.95E-02
sprouting angiogenesis	0.83	43	1.05E-02	9.10E-02
fatty-acyl-CoA metabolic process	0.91	20	1.10E-02	9.37E-02

Table S6 Motif search of predicted TFs binding motifs within DM-associated SEs.

TF	SEs (%)	Background (%)	q-value	difference of binding
STAT2	100	2.91	0	97.09
KLF4	83.36	71.34	4.86E-13	12.01
E2F6	91.49	80.52	2.05E-15	10.97
SP3	99.93	97.69	3.54E-08	2.24
WT1	100	98.06	6.26E-08	1.94
KLF5	99.93	98.13	1.38E-06	1.79
SP1	100	98.36	9.23E-07	1.64
EGR1	99.78	98.36	2.53E-04	1.42
SOX10	0.22	0	3.39E-01	0.22
SOX9	0.22	0	3.39E-01	0.22
RUNX2	0.22	0.07	7.42E-01	0.15
MITF	0.07	0	1	0.07
TP53	0.22	0.15	1	0.07
TEAD2	2.39	2.61	8.99E-01	-0.22
NFYA	13.73	14.55	7.34E-01	-0.82
PPARG	4.10	6.64	7.44E-03	-2.54
ESR1	2.84	10.22	6.93E-14	-7.39
E2F1	1.04	8.66	6.80E-19	-7.61
MZF1	1.94	25.30	3.89E-68	-23.36

Table S7 Motif search of predicted TFs binding motifs within DM-associated cooperative ENHs.

TF	Cooperative ENHs (%)	Background (%)	q-value	difference of binding
EGR1	69.28	66.23	6.98E-07	3.05
ETV4	5.70	9.96	1.47E-34	-4.26
MZF1	2.41	5.32	1.90E-31	-2.90
TP53	0.05	0	4.68E-02	0.05
TFAP2A	0.07	0.02	7.84E-02	0.06
MITF	0.01	0	1	0.01
